# Sex‐specific differences in colorectal cancer: A multicenter retrospective cohort study

**DOI:** 10.1002/cnr2.1845

**Published:** 2023-06-22

**Authors:** Hyun Jin Joo, Hyun Seok Lee, Byung Ik Jang, Dae Bum Kim, Jae Hyun Kim, Jae Jun Park, Hyun Gun Kim, Il Hyun Baek, Jun Lee, Bun Kim

**Affiliations:** ^1^ Division of Gastroenterology, Department of Internal Medicine Chung‐Ang University Medical Center Seoul Republic of Korea; ^2^ Department of Internal Medicine, School of Medicine, Kyungpook National University Kyungpook National University Hospital Daegu Republic of Korea; ^3^ Department of Internal Medicine Yeungnam University College of Medicine Daegu Republic of Korea; ^4^ Department of Internal Medicine, St. Vincent's Hospital, College of Medicine The Catholic University of Korea Suwon Republic of Korea; ^5^ Department of Internal Medicine Kosin University College of Medicine Busan Republic of Korea; ^6^ Department of Internal Medicine and Institute of Gastroenterology Yonsei University College of Medicine Seoul Republic of Korea; ^7^ Institute for Digestive Research Soonchunhyang University College of Medicine Seoul Republic of Korea; ^8^ Department of Gastroenterology, International St. Mary's Hospital Catholic Kwandong University Incheon Republic of Korea; ^9^ Department of Internal Medicine Chosun University Gwangju Republic of Korea; ^10^ Center for Colorectal Cancer and Department of Internal Medicine, Research Institute and Hospital National Cancer Center Goyang Republic of Korea

**Keywords:** colorectal neoplasm, prognosis, sex, sex characteristics, treatment

## Abstract

**Background:**

Due to sex‐specific differences in the incidence and clinical and histopathological characteristics of colorectal cancer (CRC), understanding the impact of sex on CRC may suggest sex‐targeted strategies for screening, treatment, and prevention, leading to improved prognosis of CRC. However, there have been few studies investigating the sex‐specific differences in CRC in the Republic of Korea. We aimed to assess sex differences in CRC in the Republic of Korea.

**Methods:**

This was a retrospective, multicenter, cohort study of patients diagnosed with CRC between January 2012 and December 2013 at nine hospitals. Patients who had an uncertain CRC stage, were diagnosed with other cancers within 5 years, had carcinoma in situ, non‐epithelial cancer, or primary cancer other than CRC, were excluded. Factors associated with overall survival or progression‐free survival were investigated using Cox regression analysis. Cumulative probability of metachronous lesions was compared using the Kaplan–Meier estimator survival analysis and we compared the survival curves of each group using a log‐rank test. Outcomes were compared using the chi‐square, Fisher's exact, or Student's *t*‐test, as appropriate.

**Results:**

Three thousand one hundred and forteen patients (1999 men, 1315 women) were included. There was no significant difference in the age at onset between men and women. The proportion of patients diagnosed through regular health check‐ups, and asymptomatic at time of diagnosis, was higher in men (48.9% men vs. 42.0% women, *p* < .001). Rectal cancers were more common in men (38.8% men vs. 31.8% women, *p* < .001). Right colon cancers were more common in women (31.4% women vs. 22.7% men, *p* < .001). *KRAS* mutations were found in 109/317 (34.4%) women and 112/480 (23.3%) men. Overall CRC survival and progression‐free survival were similar in both sexes.

**Conclusion:**

Sex differences in CRC may be due to the biological and social‐behavioral differences between the sexes. They should be considered during screening, diagnosis, and treatment of CRC for better outcomes.

## 1 INTRODUCTION

Colorectal cancer (CRC) is the fourth most common cancer and the third most common cause of cancer‐related deaths in the Republic of Korea.^1^ Sex has been found to be one of the most important factors underlying the incidence and mortality of CRC. In the Republic of Korea, colorectal cancer is more frequent in men, with age‐standardized rates of 38.6/100 000 for men compared to 21.8 per 100 000 for women in 2018. The mortality rate of CRC is significantly higher in men than in women.^2^

In addition to the incidence, there are sex‐related differences in clinical and histopathological characteristics, such as the morphology and anatomic location of tumors, which have been demonstrated in previous studies. Women have a higher risk of developing right‐sided CRC than men,^3^ as well as proximal colonic tumors, which are more often flat in shape, making them more difficult to distinguish by colonoscopy.^4^ These differences may explain why the clinical outcomes of CRC are dissimilar according to the sex.

Therefore, understanding the impact of sex on CRC may aid in formulating sex‐targeted strategies for screening, treatment, and prevention of CRC, which can improve the prognosis of CRC. However, scanty studies have investigated the sex‐specific differences in CRC that cover most regions of the Republic of Korea. Hence, our study aimed to accurately identify the sex differences in CRC by comparing the characteristics of CRC between men and women with CRC in the Republic of Korea.

## 2 MATERIALS AND METHODS

### 2.1 Study design and patients

This was a retrospective, multicenter, cohort study that analyzed information from electronic records of nine hospitals. Of nine hospitals, two are located in Seoul and two in Gyeonggi‐do. Except for the four hospitals located in the metropolitan area, five hospitals are located in the cities of Gyeongsang‐do, Jeolla‐do, and Chungcheong‐do. The study was reviewed and approved by the National Cancer Center Institutional Review Board (NCC2021‐0363).

**FIGURE 1:**
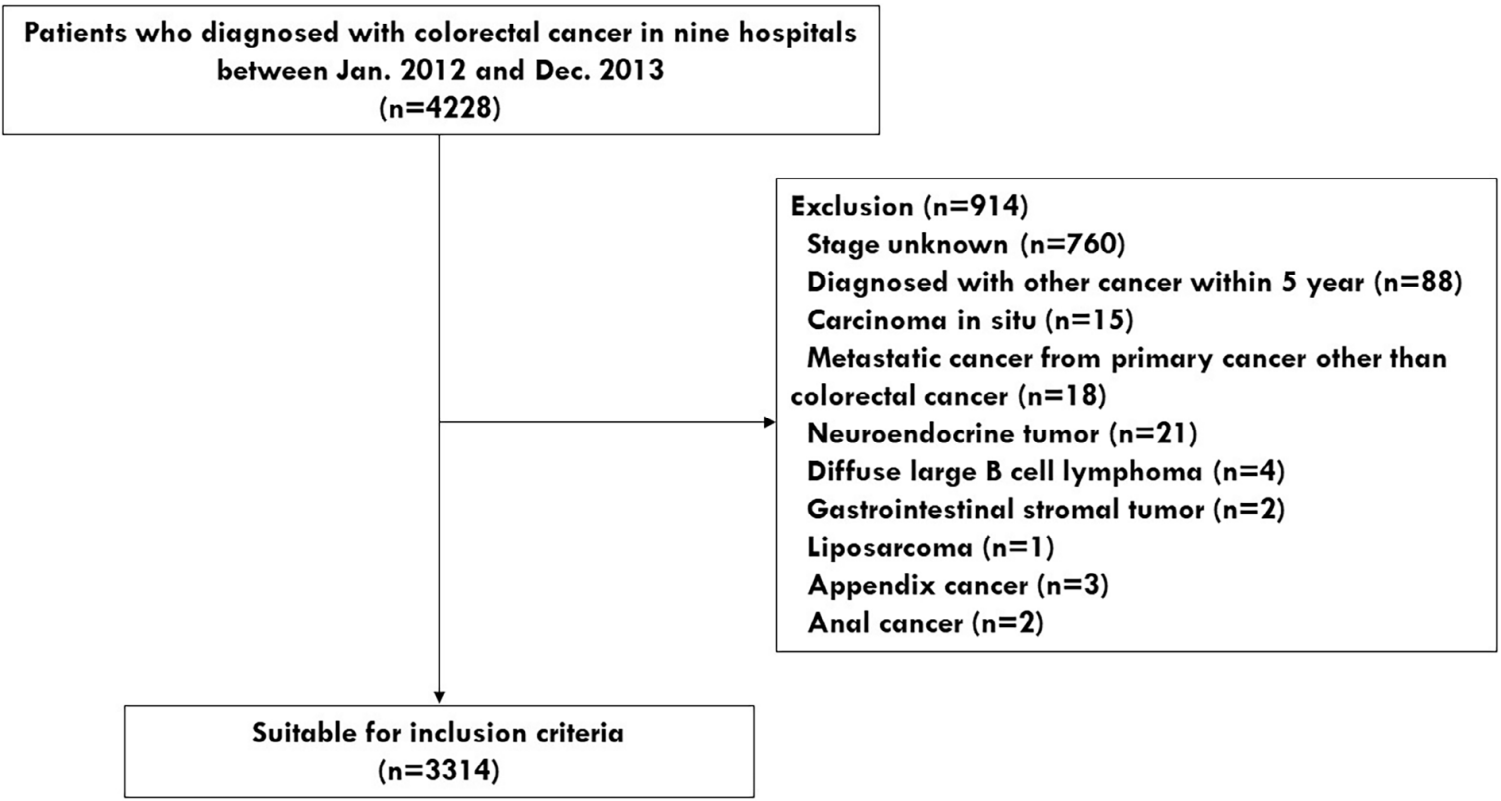
Flow diagram of patient enrollment.

The patient enrollment flow diagram is shown in Figure 1. Initially, a total of 4228 patients with CRC were recruited between January 2012 and December 2013 from nine hospitals. Only patients who were followed up for more than 6 months were enrolled. Exceptionally, patients whose follow‐up was terminated within 6 months of diagnosis of CRC due to death were enrolled in the study. Participants who met at least one of the following criteria were excluded (*n* = 914): Patients who had an uncertain CRC stage (*n* = 760), patients who were diagnosed with other cancers within 5 years prior to CRC diagnosis (*n* = 88), patients who had carcinoma in situ (*n* = 15), metastatic cancer from primary cancer other than CRC (*n* = 18), neuroendorine tumor (*n* = 21), diffuse large B cell lymphoma (*n* = 4), gastrointestinal stromal tumor (*n* = 2), liposarcoma (*n* = 1), appendix cancer (*n* = 3), or anal cancer (*n* = 2). As a result, 3314 patients (1999 men, 1315 women) were eventually included in this study.

### 2.2 Definition

Family history of CRC was defined as the diagnosis of CRC in at least one first‐degree relative. Tumor staging was performed according to the American Joint Commission on Cancer standards contemporaneous with the treatment period. Obesity was defined as body mass index ≥25 kg/m^2^ according to the cut‐off value for Asians.^5^ Patients who quit smoking for more than 1 year and those who did not smoke were referred to as ex‐ and never‐smokers, respectively. All current smokers and ex‐smokers smoked for more than 10 pack‐years.

Tumor location was categorized as the right colon (cecum, ascending colon, transverse colon), left colon (descending colon, sigmoid colon), or rectum.

### 2.3 Statistical analysis

Data were expressed as mean (±standard deviation), numbers, or numbers (%), as appropriate. Factors associated with overall survival or progression‐free survival (age, sex, smoking history, alcohol history, Eastern Cooperative Oncology Group (ECOG), body mass index (BMI), main symptom, duration of symptom, number of tumor, location of tumor, histology, histologic grade, stage of cancer, presence of immunohistochemistry markers) were investigated using Cox regression analysis. Any variables identified as significant (*p* < .05) in the univariate analysis were included in the multivariate analysis.

Cumulative probability of metachronous lesions was compared using the Kaplan–Meier estimator survival analysis, and the survival curves of each group were compared using a log‐rank test. Outcomes were compared using the chi‐square test or Fisher's exact test for categorical data and Student's *t*‐test for continuous variables (age, and duration of symptom). Data analysis was performed using SPSS software (version 14.0; SPSS Inc., Chicago, Illinois, USA). A two‐sided *p* value of <.05, was considered statistically significant.

## 3 RESULTS

### 3.1 Baseline clinical, pathological, and molecular characteristics

A total of 1999 women and 1315 men were included in this study, with a total study population of 3114. The demographic, clinical, histologic, and pathological characteristics of the patients analyzed according to the sex are shown in Tables 1, 2, 3.

**TABLE 1:** Clinical characteristics of the patients.

Patient characteristics	Men (*n* = 1999)	Women (*n* = 1315)	Total (*n* = 3314)	*p* Value
Age of onset, years	64.5 ± 11.3	65.2 ± 13.1	64.8 ± 12.0	.138
Smoking history				<.001
Non smokers	1063 (53.2%)	1247 (94.8%)	2310 (69.7%)	
Current smokers, past smokers	936 (46.8%)	68 (5.2%)	1004 (30.3%)	
Alcohol history				<.001
Non drinker	1022 (51.5%)	1163 (88.9%)	2185 (66.3%)	
Drinker	964 (48.5%)	145 (11.1%)	1109 (33.7%)	
ECOG				.145
0	838 (65.9%)	581 (64.1%)	1419 (65.2%)	
1	399 (31.4%)	282 (31.1%)	681 (31.3%)	
2	24 (1.9%)	33 (3.6%)	57 (2.6%)	
3	8 (0.6%)	7 (0.8%)	15 (0.7%)	
4	3 (0.2%)	3 (0.3%)	6 (0.3%)	
BMI ≥ 25	554 (29.3%)	364 (29.1%)	918 (29.2%)	.940
Main symptom				<.001
No symptom	950 (48.9%)	532 (42.0%)	1482 (46.2%)	
General weakness	88 (4.5%)	54 (4.3%)	142 (4.4%)	
Abdominal pain	393 (20.2%)	267 (21.1%)	660 (20.6%)	
Hematochezia	200 (10.3%)	138 (10.9%)	338 (10.5%)	
Melena	12 (0.6%)	5 (0.4%)	17 (0.5%)	
Constipation	10 (0.5%)	16 (1.3%)	26 (0.8%)	
Duration of symptom, days	109.4 ± 405.0	96.2 ± 240.8	103.8 ± 344.9	.448

Abbreviations: BMI, body mass index; ECOG, Eastern Cooperative Oncology Group.

**TABLE 2:** Pathological characteristics of the patients.

Tumor characteristics	Number of cases (proportion of cases)			*p* Value
	Men (*n* = 1999)	Women (*n* = 1315)	Total (*n* = 3314)	
Number of tumors				.015
1	1905 (96.7%)	1279 (98.4%)	3184 (97.3%)	
2	63 (3.2%)	19 (1.5%)	82 (2.5%)	
3	2 (0.1%)	2 (0.2%)	4 (0.1%)	
4	1 (0.1%)	0 (0.0%)	1 (0.0%)	
Location of the tumor				<.001
Right colon	448 (22.7%)	407 (31.4%)	855 (26.1%)	
Left colon	758 (38.4%)	478 (36.8%)	1236 (37.8%)	
Rectum	766 (38.8%)	413 (31.8%)	1179 (36.1%)	
Histology				.416
Adenocarcinoma WD	368 (18.4%)	232 (17.6%)		
Adenocarcinoma MD	1494 (74.7%)	970 (73.8%)		
Adenocarcinoma PD	85 (4.3%)	68 (5.2%)		
Mucinous carcinoma	46 (2.3%)	46 (2.3%)		
Signet ring cell	6 (0.3%)	5 (0.4%)		
Squamous cell carcinoma	0 (0.0%)	1 (0.1%)		
Histologic grade				.08
Low	680	404		
High	140	102		
T stage				.112
1	297 (16.3%)	192 (15.9%)	489 (16.1%)	
2	243 (13.3%)	170 (14.0%)	413 (13.6%)	
3	1016 (55.7%)	634 (52.4%)	1650 (54.3%)	
4	269 (14.7%)	215 (17.8%)	484 (15.9%)	
N stage				.126
0	1032 (56.1%)	648 (52.3%)	1680 (54.6%)	
1	487 (26.5%)	351 (28.4%)	838 (27.2%)	
2	320 (17.4%)	238 (19.2%)	558 (18.1%)	
3	0 (0.0%)	1 (0.1%)	1 (0.0%)	
M stage				.484
1	1544 (87.2%)	1031 (86.3%)	2575 (86.8%)	
2	226 (12.8%)	164 (13.7%)	390 (13.2%)	
TNM stage of cancer				.207
1	403 (23.0%)	281 (23.8%)	684 (23.3%)	
2	522 (29.8%)	309 (26.2%)	831 (28.3%)	
3	604 (34.4%)	429 (36.3%)	1033 (35.2%)	
4	225 (12.8%)	162 (13.7%)	387 (13.2%)	

Abbreviations: MD, moderately differentiated carcinoma; PD, poorly differentiated; WD, well‐differentiated carcinoma.

**TABLE 3:** Molecular characteristics.

Immunohistochemistry markers	Number of positive cases (proportion of positive cases)			*p* Value
	Men	Women	Total	
hMLH1 (*n* = 1897)	929 (81.2%)	929 (81.2%)	1533 (80.8%)	.632
hMSH2 (*n* = 1897)	957 (83.7%)	632 (83.8%)	1589 (83.8%)	1
hMSH6 (*n* = 878)	440 (81.3%)	267 (79.2%)	707 (80.5%)	.498
hPMS2 (*n* = 868)	428 (79.4%)	248 (75.4%)	676 (77.9%)	.193
MSI (*n* = 685)				.156
MSS, MSI‐L	403 (97.8%)	261 (95.6%)	664 (96.9%)	
MSI‐H	9 (2.2%)	12 (4.4%)	21 (3.1%)	
*KRAS* (*n* = 797)	112 (23.3%)	109 (34.4%)	221 (27.7%)	.001
NRAS (*n* = 173)	2 (1.9%)	1 (1.5%)	3 (1.7%)	1
HRAS (*n* = 158)	0 (0.0%)	1 (1.5%)	1 (0.6%)	.857
BRAF (*n* = 285)	1 (0.6%)	1 (0.9%)	2 (0.7%)	1

There was no significant difference in age at onset between men and women. Smokers (46.8% men vs. 5.2% women, *p* < .001) and alcohol drinkers (48.5% men vs. 11.1% women, *p* < .001) were more frequent among men. The proportion of asymptomatic patients at the time of diagnosis, who were diagnosed through regular health check‐ups, was higher in men than that in women (48.9% men vs. 42.0% women, *p* < .001) (Table 1).

Synchronous cancer was more frequent in men than in women (3.3% men vs. 1.6% women, *p* = .015). Rectal cancers were more common in men than in women (38.8% men vs. 31.8% women, *p* < .001), whereas right colon cancers were more common in women than in men (31.4% women vs. 22.7% men, *p* < .001). However, the histology and proportions of cancer stages at the time of diagnosis were similar for both sexes (Table 2).

*KRAS* mutations were found in 109 (34.4%) of 317 women and 112 (23.3%) of 480 men (*p* = .001). *MSI* status and *BRAF* mutations were similar in both sexes (Table 3).

### 3.2 Treatment according to the sex

Overall, 88.1% of men and 88.2% of women with CRC underwent curative treatment. Surgery was the most common initial treatment in both sexes. However, more men received concurrent chemo‐radiotherapy before resection than women (8.8% men vs. 5.9% women, *p* = .033) (Table 4). In rectal cancer patients, there was no significant difference between sexes in patients who underwent pre‐operative CRT (male: 22.4% vs. female: 18.2%, *p* = .338).

**TABLE 4:** Treatment.

Treatment	Number of cases (proportion of cases)		*p* Value
	Men (*n* = 1999)	Women (*n* = 1315)	
Curative treatment	1372 (88.1%)	904 (88.2%)	0.783
Palliative treatment	185 (11.9%)	121 (11.8%)	
Initial treatment			0.637
Endoscopic resection	209 (18.4%)	147 (18.3%)	
Operation only	463 (40.7%)	333 (40.5%)	
Operation + chemotherapy	359 (31.6%)	289 (35.2%)	
Operation + chemo‐radiotherapy	49 (4.3%)	20 (2.4%)	
Chemotherapy	39 (3.4%)	20 (2.4%)	
Chemo‐radiotherapy	9 (0.8%)	4 (0.5%)	
Supportive care	6 (0.5%)	9 (1.1%)	
Pre‐operative treatment			0.033
Chemotherapy only	99 (5.9%)	60 (5.4%)	
Radiotherapy only	2 (0.1%)	2 (0.2%)	
Chemo‐radiotherapy	149 (8.8%)	66 (5.9%)	
Post‐operative chemotherapy	875 (51%)	591 (52.6%)	0.406
Post‐operative radiotherapy	73 (4.8%)	37 (3.7%)	0.216
Pre‐operative chemo‐radiotherapy in rectal cancer patients	172 (22.4%)	75 (18.1%	0.338

### 3.3 Survival according to the sex

**FIGURE 2:**
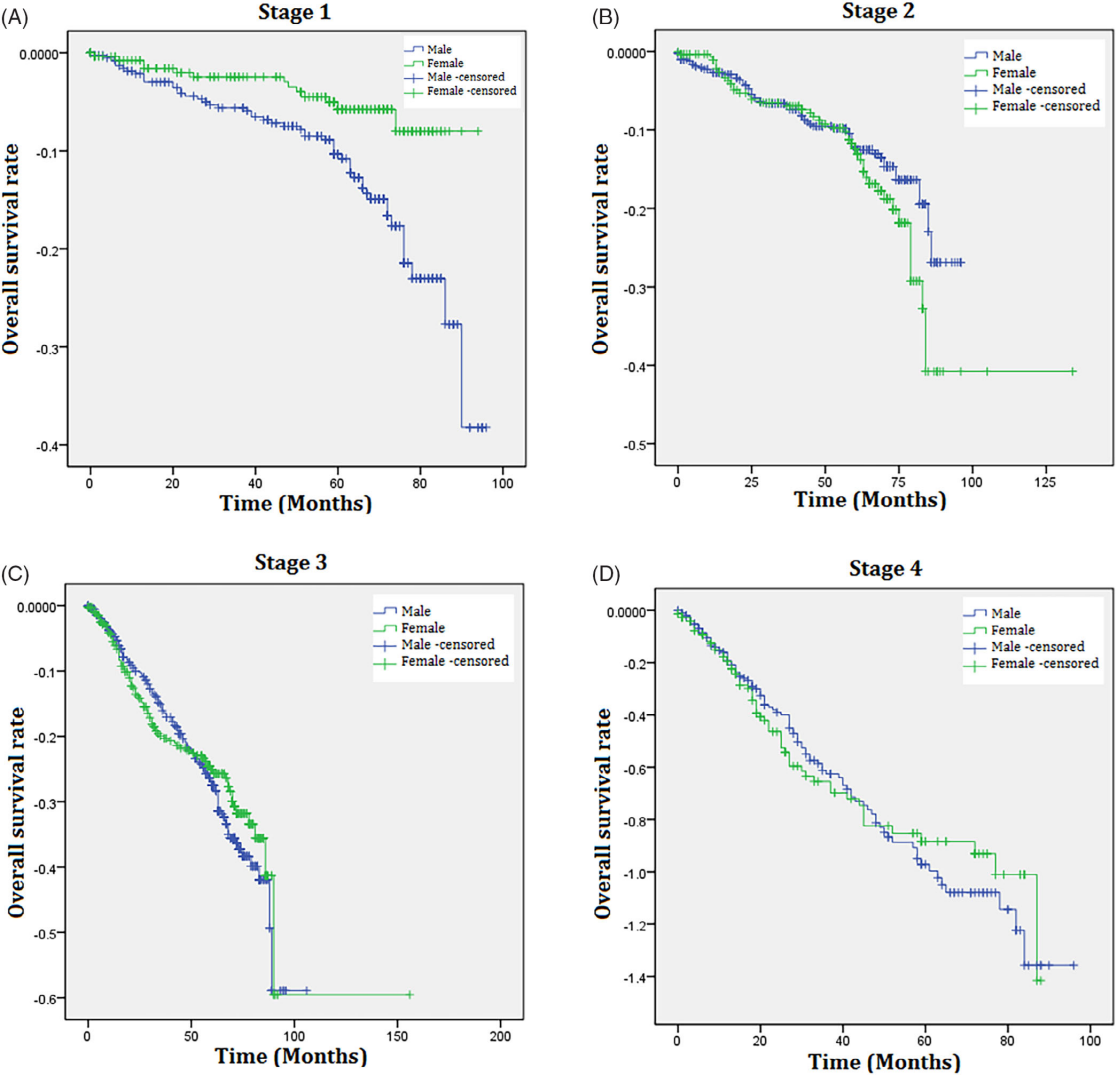
Kaplan–Meier survival plots for overall survival of men and women with colorectal cancer. (A) Stage I, (B) Stage II, (C) Stage III, (D) Stage IV.

**FIGURE 3:**
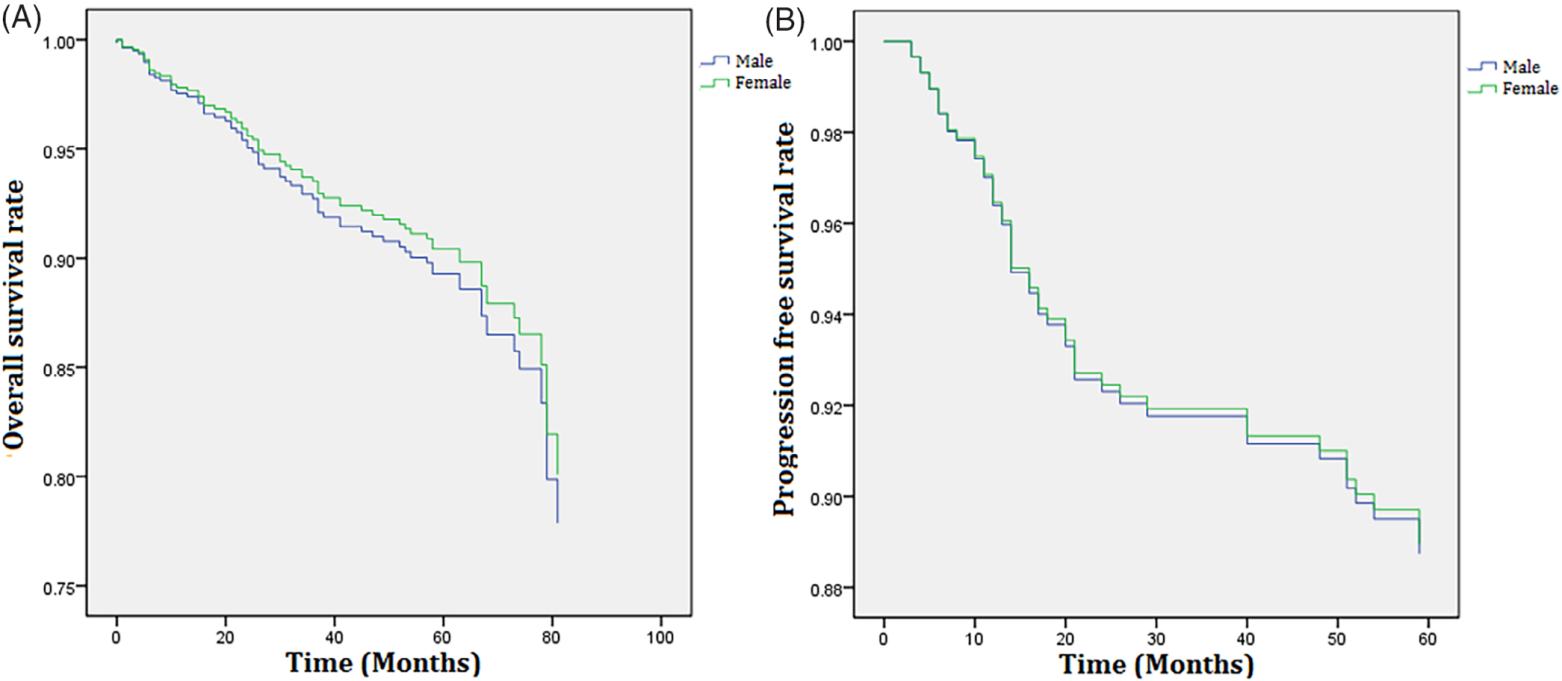
Cox regression survival plots of men and women with colorectal cancer showing the (A) Overall survival rate, (B) Progression‐free survival rate.

According to the results of the Kaplan–Meier survival analysis, women showed a higher overall survival rate in Stage I compared to men, whereas there were no sex differences in the overall survival in stages II, III, and IV (Figure 2). Subsequently, the results of multivariate Cox regression analysis, which included age, sex, smoking history, alcohol history, ECOG, stage of cancer, location of cancer, and the presence of *KRAS* mutations and MSI, revealed that overall survival and progression‐free survival were similar for both sexes (Figure 3).

## 4 DISCUSSION

This retrospective cohort study conducted a sex focused analysis of multicenter data on CRC in the Republic of Korea. Several differences were revealed between men and women with CRC, potentially related to screening, diagnosis, and treatment.

According to national cancer statistics, the incidence of CRC among men was significantly higher than that among women in the Republic of Korea.^1^ This sex difference in the overall CRC incidence was observed with a similar sex ratio in our data.

Male sex has been proposed to be a risk factor for CRC because of many biological and behavioral factors.^6^, ^7^, ^8^ For example, men have higher alcohol consumption and smoking rates^9^, ^10^ along with a greater propensity to deposit visceral fat,^11^ leading to an increased risk of CRC, compared to women.^12^, ^13^, ^14^ Moreover, Several previous studies have suggested that testosterone may promote colorectal neoplasm formation.^15^, ^16^ And estrogen has been reported to play a protective role against incidence of CRC, which may be a reason for the relatively low CRC incidence in women.^17^

Up to now, there have been many studies showing that the microbiome plays a profound role in development, progression and treatment response of colon cancer.^18^, ^19^ Also, it is well known that the distribution of gut microbiota differs according to sex.^20^ Therefore, the microbiome is a possible key cause of differences between men and women in CRC.

The proportion of patients who were diagnosed through regular health check‐ups, and were asymptomatic at the time of diagnosis, was higher among men compared to women. However, there was no difference between the sexes in the time taken to receive medical treatment after the onset of symptoms. It is known that men are generally less aware of cancer signs and symptoms, such as recognizing changes in the bowel habit.^21^, ^22^, ^23^, ^24^, ^25^ However, even if women are more sensitive to CRC symptoms, they usually face a greater barrier to consulting a doctor. Therefore, this does not mean that they have a shorter delay from the onset of symptoms until the first medical consultation.^26^, ^27^, ^28^

Our study reported that there were no significant differences by sex in the initial treatment methods for CRC. A Swiss study about sex differences in CRC treatment revealed that there was no sex difference in treatment decisions, and that the probability of receiving initial treatment other than surgery was higher in patients with comorbidities than in patients without comorbidities, and this effect was stronger in women than in men.^29^

*KRAS* mutations were found in 109 (34.4%) of 317 women and 112 (23.3%) of 480 men with CRC. Women were also found to have *KRAS* mutations in codon 12 more frequently than men, which are associated with more advanced adenomas.^30^

Several studies reported that a higher proportion of women presented with right‐sided CRC than men, which is consistent with our findings.^24^, ^31^ In a study about colorectal cancer risk of type 2 diabetes patients, the risk of developing distal colon cancer was higher in men, and the risk of developing proximal colon cancer was higher in female.^32^ Colon cancer has different molecular and pathological characteristics according to the tumor location. Right‐sided colon cancer is more advanced and less differentiated than left‐sided colon cancer.^26^ Endoscopic exams have also clearly revealed the morphological differences between right and left colon cancers. A higher proportion of women showed flat and depressed‐type CRC lesions, while a higher proportion of men demonstrated polypoid‐type lesions, which are more easily detected.^4^ Patients with right colon cancer exhibited more ambiguous symptoms.^26^ Furthermore, women were found to have a longer transverse colon and higher redundancy compared to men. These sex‐specific anatomical and physiological characteristics may result in incomplete colonoscopy in women, making it challenging to detect tumors in endoscopies.^27^ These findings suggest that more attention needs to be given to colon cancer screening in women in terms of sensitivity. Additionally, sex‐specific screening guidelines for CRC are required.

Overall survival and progression‐free survival were similar in both sexes in our cohort although women with stage I CRC showed better overall survival than men with stage I CRC. This finding may be partly explained by the better survival of women in the general population because non‐cancer deaths have a significant impact on the survival rates of patients with stage I cancer.

A few studies have reported that women have worse survival than men.^28^, ^29^ These differences were particularly significant among older patients. According to previous studies, economic difficulties were associated with a reluctance to undergo colon cancer screening,^28^ and older women with advanced‐stage cancer were found to behave passively while undergoing aggressive medical therapy.^30^ These patients are also less likely to undergo regular medical check‐ups, and may have fewer opportunities for early diagnosis. Therefore, the poor survival rate in older women may be associated with socioeconomic factors. Perhaps, if women and men had equal opportunities for screening and treatment, there would be no sex differences in the survival rate of patients with CRC.

The major strength of this study is that our multicenter data could represent the population of most regions in the republic of Korea. However, there are several limitations. First, this is a retrospective study, which is subject to potential bias. Second, there is lack of gene mutation data. Although we have large study population, the results of genetic mutation were not obtained from all subjects, and the sample size was small in some variables. However, the incompleteness is unlikely to vary by sex. Further study with sufficient sample sized is needed to understand sex differences in CRC that focus on gene mutation.

## 5 CONCLUSION

In conclusion, this study shows that there are sex‐specific differences such as location of cancer, frequency of gene mutation, and survival rate of stage I cancer in patients with CRC. Biological and social‐behavioral differences between men and women seem to be the reason for the differences in the characteristics of CRC. The results of this study imply that there may be sex‐specific differences in optimal colon cancer diagnosis and treatment. Further studies on sex‐specific differences in comprehensive molecular analysis and treatment response are required.

## AUTHOR CONTRIBUTIONS

**Hyun Jin Joo:** Formal analysis (lead); writing – original draft (lead). **Hyun Seok Lee:** Data curation (equal); resources (equal). **Byung Ik Jang:** Data curation (equal); resources (equal). **Dae Bum Kim:** Data curation (equal); resources (equal). **Jae Hyun Kim:** Data curation (equal); resources (equal). **Jae Jun Park:** Data curation (equal); resources (equal). **Hyun Gun Kim:** Data curation (equal); resources (equal). **Il Hyun Baek:** Data curation (equal); resources (equal). **Bun Kim:** Conceptualization (lead); supervision (lead); writing – review and editing (lead). **Jun Lee:** Data curation (equal).

## CONFLICT OF INTEREST STATEMENT

The authors have stated explicitly that there are no conflicts of interest in connection with this article.

## ETHICS STATEMENT

The study was reviewed and approved by the National Cancer Center Institutional Review Board (NCC2021‐0363).

## Data Availability

The data that support the findings of this study are available from the corresponding author upon reasonable request.
